# The Number and Position of Orai3 Units within Heteromeric Store-Operated Ca^2+^ Channels Alter the Pharmacology of I_CRAC_

**DOI:** 10.3390/ijms21072458

**Published:** 2020-04-02

**Authors:** Sven Kappel, Tatiana Kilch, Roland Baur, Martin Lochner, Christine Peinelt

**Affiliations:** 1 Institute of Biochemistry and Molecular Medicine, University of Bern, Bühlstrasse 28, 3012 Bern, Switzerland; sven.kappel@ibmm.unibe.ch (S.K.); roland.baur@ibmm.unibe.ch (R.B.); martin.lochner@ibmm.unibe.ch (M.L.); 2 Auf der Schled 19, 66822 Lebach, Germany; tatiana.kilch@gmx.net

**Keywords:** Orai1, Orai3, concatenated channels, I_CRAC_, SOCE, 2-APB

## Abstract

Store-operated heteromeric Orai1/Orai3 channels have been discussed in the context of aging, cancer, and immune cell differentiation. In contrast to homomeric Orai1 channels, they exhibit a different pharmacology upon application of reactive oxygen species (ROS) or 2-aminoethoxydiphenyl borate (2-APB) in various cell types. In endogenous cells, subunit composition and arrangement may vary and cannot be defined precisely. In this study, we used patch-clamp electrophysiology to investigate the 2-APB profile of store-operated and store-independent homomeric Orai1 and heteromeric Orai1/Orai3 concatenated channels with defined subunit compositions. As has been shown previous, one or more Orai3 subunit(s) within the channel result(s) in decreased Ca^2+^ release activated Ca^2+^ current (I_CRAC_). Upon application of 50 µM 2-APB, channels with two or more Orai3 subunits exhibit large outward currents and can be activated by 2-APB independent from storedepletion and/or the presence of STIM1. The number and position of Orai3 subunits within the heteromeric store-operated channel change ion conductivity of 2-APB-activated outward current. Compared to homomeric Orai1 channels, one Orai3 subunit within the channel does not alter 2-APB pharmacology. None of the concatenated channel constructs were able to exactly simulate the complex 2-APB pharmacology observed in prostate cancer cells. However, 2-APB profiles of prostate cancer cells are similar to those of concatenated channels with Orai3 subunit(s). Considering the presented and previous results, this indicates that distinct subtypes of heteromeric SOCE channels may be selectively activated or blocked. In the future, targeting distinct heteromeric SOCE channel subtypes may be the key to tailored SOCE-based therapies.

## 1. Introduction

In the last 14 years, many different groups advanced the understanding of structure–function relationships of store-operated Ca^2+^ entry (SOCE) channels. Numerous extracellular stimuli produce intracellular second messenger inositol 1,4,5-trisphosphate (IP_3_) that subsequently releases Ca^2+^ via IP_3_ receptors from intracellular Ca^2+^ stores, i.e., the endoplasmic reticulum (ER). Upon ER depletion, Ca^2+^ dissociates from stromal interaction molecule 1′s (STIM1) EF hand motif in the ER lumen. Consequently, STIM1 cluster in the ER membrane recruit and activate Orai1 channels in the plasma membrane. Studies in the past proposed a tetramer of Orai1 proteins to form a functional SOCE channel [1,2,3,4]. Later on, functional and structural studies suggested hexameric Orai1 channels [5,6,7]. In endogenous cells, large Orai1 channel complexes have been observed in liquid-phase scanning transmission electron microscopy and in native gels that may have been of tetrameric or hexameric origin [8]. The number of STIM1 molecules per Orai1 subunit varies and allows for a graded activation of SOCE with an optimal STIM1:Orai1 ratio of 2:1 [9,10,11,12].

Apart from Orai1, Orai2 and Orai3 isoforms are expressed throughout various tissues [13]. In addition to forming homomeric Orai1 SOCE channels, Orai1 proteins multimerize with Orai family members Orai2 and Orai3 when overexpressed [14]. Endogenous heteromeric SOCE channels have been reported in different cell types [15,16,17,18,19,20,21,22]. Orai3 may be a negative modulator as, upon knockdown of Orai3, SOCE can be increased [15,16,17,18,23,24]. In contrast, in other studies, SOCE is decreased upon knockout of Orai3, indicating a role as a positive modulator of SOCE [25,26,27,28]. In addition, heteromeric Orai1/Orai3 channels can be activated store-independently [29,30,31] and/or form arachidonic acid-activated pentameric channels [32,33]. Finally, Orai3 has also been reported to form ER Ca^2+^ leakage channels [34]. Many pathophysiological cellular functions in aging and cancer involve SOCE and Orai3 [26,27,35,36,37,38,39,40], highlighting the importance of understanding the pharmacology of Orai1/Orai3 heteromeric SOCE channels.

A widely used tool compound to investigate SOCE and the underlying Ca^2+^ release activated Ca^2+^ current (I_CRAC_) is 2-aminoethoxydiphenyl borate (2-APB). Upon application of 2-APB, I_CRAC_ is first amplified and then blocked in different cell types, including T cells, B cells and mast cells [41,42,43]. One hallmark of the STIM1/Orai1-mediated I_CRAC_ is its bimodal action upon application of 50 µM 2-APB: I_CRAC_ is first amplified and then blocked [44]. The 2-APB response depends on the STIM1/Orai1 ratio [45]. The gathered data suggest that 2-APB’s action is directed toward the STIM1-Orai1 coupling interface [46] and I_CRAC_ amplification is due to Orai1 pore dilation [47] and/or enhancement of STIM1/Orai1 coupling [48]. I_CRAC_ mediated by STIM1/Orai2 and STIM1/Orai3 channels differs in its response upon application of 50 µM 2-APB. In STIM1/Orai2 cells, 50 µM 2-APB first amplifies and then blocks I_CRAC_ by ~ 50% whereas I_CRAC_ mediated by STIM1/Orai3 is not blocked but amplified [14]. In addition, upon the application of 2-APB, STIM1/Orai3-mediated I_CRAC_ develops a large outward current and can be activated by 2-APB without STIM1 [49,50,51], presumably due to a widening of the pore [52].

First structural insights regarding the differences between Orai1 and Orai3-containing channels was reported by Zhang et al. Using various chimeric Orai1/Orai3 constructs, they demonstrated that the segment between the second and third transmembrane domain is responsible for 2-APB-induced Orai3 currents [51]. Later on, residues C101 and G158 within this sequence were proposed to be close to the 2-APB-sensitive site [53] and the 2-APB-induced pore is formed by TM1 residues and E165 in TM3 [54]. Furthermore, the N-terminus of Orai3 was suggested to be involved in 2-APB-dependent gating of Orai3 [55]. Conferring the N-terminus of Orai3 to Orai1 also decreased affinity for scaffold protein AKAP79 [56]. It has been proposed that before 2-APB is able to gate Orai3, STIM1 has to dissociate from the channel complex first [57]. Experiments with Orai1-Orai3 tandem constructs demonstrated that heteromeric store-operated Orai1/Orai3 channels exhibit a reduced Ca^2+^ selectivity and a pharmacological profile that differs from homomeric Orai1 or Orai3 channels [58].

The 2-APB-induced response in endogenous systems varies with Orai1 and Orai3 expression levels [18,25,27,28,40,59]. Tetrameric concatenated Orai channels have been shown to mediate I_CRAC_ in the past [33]. For instance, these channel constructs have been used to demonstrate that one Orai3 subunit within heterotetrameric concatenated Orai1/Orai3 channels confers insensitivity against reactive oxygen species (ROS) to I_CRAC_ [15]. Deciphering the multiplex pharmacological profile of heteromeric CRAC channels could be the key towards targeting specific CRAC channel subtypes in diseases. With this in mind, we investigated 2-APB pharmacology of tetrameric concatenated Orai1 and Orai1/Orai3 channels.

## 2. Results

### 2.1. Store-Operated Orai1, Orai3 and Concatenated Orai1/Orai3 Channels

**Figure 1:**
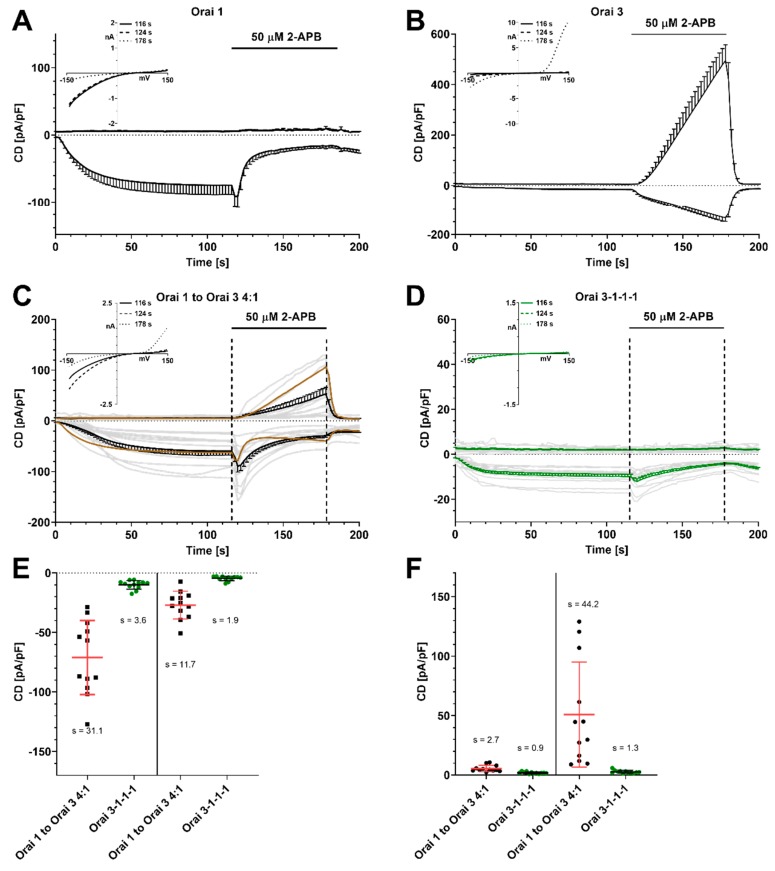
2-APB-mediated current density (CD) responses in HEK cells stably overexpressing STIM1 with 50 μM IP_3_ and 0 nM Ca^2+^ in the patch pipette. (**A**) CD development over time with Orai1 overexpression (*n* = 8) and application of 50 μM 2-APB as indicated. Inset: corresponding IV relationship. (**B**) Same experimental setting as in (A) but with Orai3 overexpression (*n* = 10). (**C**) Same experiment as in (A) but with overexpression of Orai1 and Orai3 in a ratio of 4:1 (*n* = 12) or (**D**) with overexpression of concatenated tetrameric channel 3-1-1-1 (*n* = 12), insets: corresponding IV relationship. The light grey traces are single cell recordings. A calculated CD taken from experiments in (A) and (B) is represented as a dark brown line in (C) (4 × CD from Orai1 + 1 × CD from Orai3 divided by 5). (**E**) Extracted inward current densities from (C) + (D) at t = 116 s and t = 178 s. Single data points and mean values ± SD are shown. In addition, SD is stated as s. (**F**) same as in (E) but with extracted outward currents at the same time points.

We transfected HEK293 cells stably expressing STIM1 (HEK STIM1) with Orai1 (Figure 1A) or Orai3 (Figure 1B) and evoked I_CRAC_ with 50 µM IP_3_ and 20 mM BAPTA in the patch pipette. Whole cell currents were extracted at -130 mV and +130 mV and plotted versus time. Upon I_CRAC_ development, we applied 50 µM 2-APB from 120–180 s. Under these conditions STIM1/Orai1-mediated I_CRAC_ is first amplified and then blocked, while STIM1/Orai3-mediated I_CRAC_ (CD at t = 116 s ~11 pA/pF) was amplified and a large outward current developed. Current–voltage relationships (IV) at the indicated time points are shown as insets (Figure 1A,B). These findings are in line with previously published results [50,60].

In order to investigate the 2-APB-induced pharmacological profile of heteromeric Orai1/Orai3 CRAC channels, we transfected HEK STIM1 with Orai1 and Orai3 with a ratio of 4:1, assuming that in most cells Orai1 is the predominant endogenously expressed family member. We evoked I_CRAC_ and applied 50 µM 2-APB as described above and CD was plotted versus time (Figure 1C). IVs extracted at the indicated time points are shown as inset (Figure 1C). Under these conditions, Orai1/Orai3-mediated I_CRAC_ is reduced compared to Orai1-mediated currents. Upon application of 2-APB, the average CD via Orai1/Orai3 (Figure 1C) is reduced to a lesser extent than Orai1 (Figure 1A) and an outward current developed that was lower when compared to Orai3-mediated currents (Figure 1B). Using our experimental conditions, cellular responses varied as cells expressed different ratios of Orai1/Orai3 subunits and within one cell, channels may have been assembled in varying ratios (light grey traces in Figure 1C). We next transfected a construct expressing a concatenated Orai1/Orai3 channel composed of one Orai3 and three Orai1 subunits (3-1-1-1) to investigate channels with a defined subunit composition and arrangement (Figure 1D, single cell responses light grey traces). Under these conditions, a reduced I_CRAC_ and no outward current developed. The IVs are shown as inset in Figure 1D. Analysis of IP_3_-induced inward currents and inward currents when 2-APB was applied extracted from single cells from Figure 1C,D is shown in Figure 1E. Both parameters display a higher variance when channels were transfected with Orai1 and Orai3 using a ratio of 4:1, compared to responses given by cells transfected with the concatenated channel (3-1-1-1). Figure 1F compares outward CD from both systems. Whilst variation of outward CD before 2-APB application is similar, outward currents of Orai1/Orai3 transfected cells show a larger scattering (s = 44.2) compared to 3-1-1-1 (s = 1.3) transfected cells.

**Figure 2:**
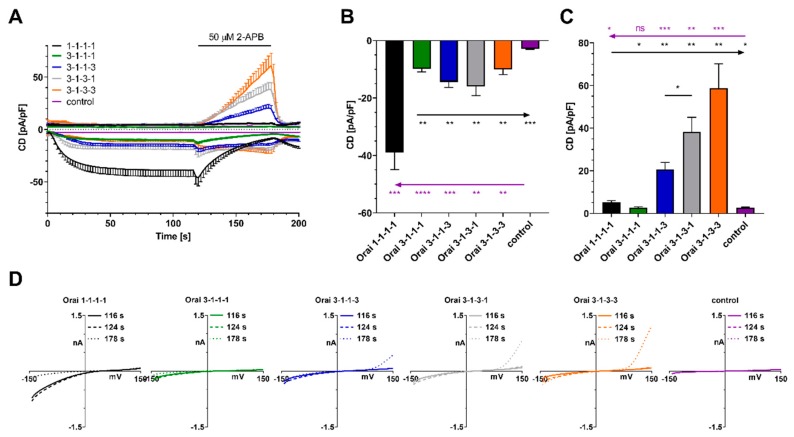
Current density responses in HEK cells stably overexpressing STIM1 with overexpression of concatenated tetrameric Orai1/Orai3 channels or empty vector control under store-depleting conditions. (**A**) CD development over time and application of 50 μM 2-APB as indicated (1-1-1-1, *n* = 6; 3-1-1-1, *n* = 12; 3-1-1-3, *n* = 10; 3-1-3-1, *n* = 7; 3-1-3-3, *n* = 10; control, *n* = 7). (**B**) Inward currents at t = 116 s extracted from (**A**). (**C**) Outward currents extracted at t = 178 s extracted from (**A**). (**D**) Corresponding IV relationships from (**A**). Statistical significance was determined by an unpaired t test with Welch’s correction and is presented as ns *p* > 0.05, * *p* < 0.05, ** *p* < 0.005, *** *p* < 0.001, **** *p* < 0.0001.

Given the variance of responses in Orai1:Orai3 (4:1) transfected cells, we assessed the pharmacological profile of 2-APB in several store-operated concatenated channel constructs (1-1-1-1, 3-1-1-1, 3-1-1-3, 3-1-3-1, 3-1-3-3) (Figure 2A). Cells expressing concatenated Orai channels display a significantly larger IP_3_-induced current compared to control transfected cells, demonstrating that store release can activate all concatenated channels (Figure 2B). In addition, all concatenated Orai channels expressing one or more Orai3 subunits display a significantly reduced IP_3_-induced current when compared to homomeric Orai1 concatenated channels (Figure 2B). These findings are in line with previous reports [15,33]. Upon application of 2-APB, concatenated channels with two or more Orai3 channels developed an outward current that increased with the number of Orai3 subunits within the construct (Figure 2C). The arrangement of Orai1 and Orai3 in the channel apparently plays a role as 3-1-3-1 displays a significantly larger outward current component than 3-1-1-3. IVs extracted at the indicated time points from cells expressing constructs 1-1-1-1, 3-1-1-1, 3-1-1-3, 3-1-3-1, 3-1-3-3 or cells that were control transfected are shown in Figure 2D.

### 2.2. Non-Store-Operated Orai1, Orai3 and Concatenated Orai1/Orai3 Channels

**Figure 3:**
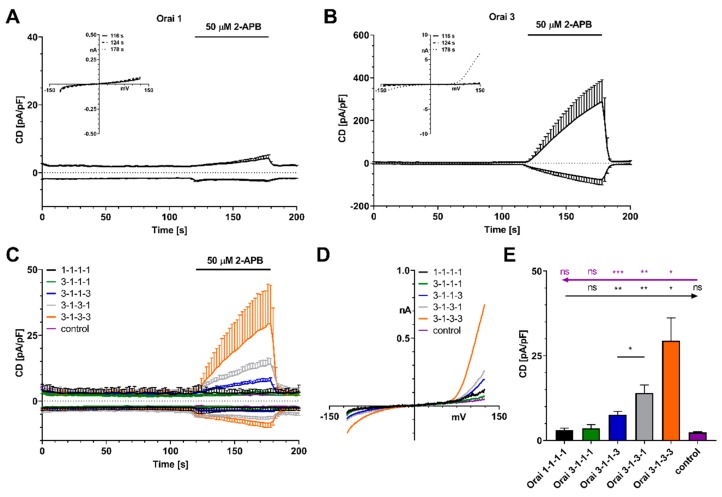
2-APB-mediated current density responses in HEK cells stably overexpressing STIM1 with internal Ca^2+^ clamped to 200 nM and no store depletion. (**A**) CD development over time with Orai1 overexpression (*n* = 9) and application of 50 μM 2-APB as indicated. Corresponding IV relationship in inset on the upper left. (**B**) Same experimental setting as in (**A**) but with Orai3 overexpression (*n* = 8). (**C**) Same experimental setting as in (**A**) but with overexpression of concatenated tetrameric Orai1/Orai3 channels or empty vector as a control (1-1-1-1, *n* = 7; 3-1-1-1, *n* = 5; 3-1-1-3, *n* = 9; 3-1-3-1, *n* = 8; 3-1-3-3, *n* = 5; control, *n* = 6). (**D**) Corresponding IV relationships extracted from cells in (**C**). (**E**) Analysis of 2-APB-mediated outward currents from (**C**) at t = 178 s. Statistical significance was determined by an unpaired t test with Welch’s correction and is indicated as ns *p* > 0.05, * *p* < 0.05, ** *p* < 0.005, *** *p* < 0.001.

We next investigated the pharmacological profile upon application of 50 µM 2-APB when HEK STIM1 cells were transfected with Orai1, Orai3 or concatenated channels (1-1-1-1, 3-1-1-1, 3-1-3-1, 3-1-1-3, 3-1-3-3) and store-depletion was prevented by omitting IP_3_ and clamping intracellular Ca^2+^ to 200 nM (Figure 3).

Using this experimental set up, 50 µM 2-APB induced a small outward current in cells expressing Orai1 (Figure 3A), while cells expressing Orai3 developed a large outward current (Figure 3B) in line with previously published results [60]. IVs at the indicated time points are displayed as insets (Figure 3A,B). We next assessed 2-APB-induced currents from cells expressing concatenated channels, when store depletion was omitted (Figure 3C). All IVs are displayed in Figure 3D and quantification of CDs is given in Figure 3E. 2-ABP induced no currents in cells expressing concatenated channels containing no or one Orai3 subunit. 2-APB-induced outward currents were observed when two or more Orai3 subunits were present in the channel and an increasing number of Orai3 channels led to increased outward currents. The order of Orai3 subunits within the concatenated channel apparently determines the size of the outward current as the current size is larger in 3-1-3-1 compared to 3-1-1-3.

**Figure 4:**
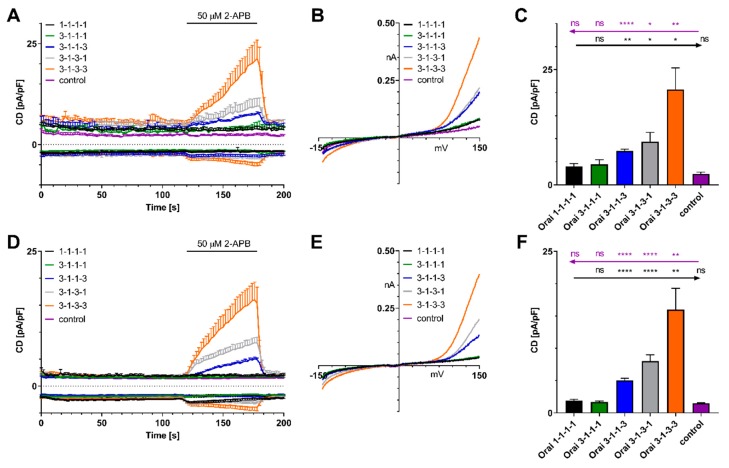
2-APB-mediated current density responses in HEK 293 WT cells. (**A**) CD development after overexpression of concatenated tetrameric Orai1/Orai3 channels or empty vector as a control when internal Ca^2+^ is clamped to 200 nM (1-1-1-1, *n* = 9; 3-1-1-1, *n* = 7; 3-1-1-3, *n* = 5; 3-1-3-1, *n* = 8; 3-1-3-3, *n* = 9; empty vector, *n* = 7). Application of 50 μM 2-APB as indicated. (**B**) Corresponding IV relationships from cells in (**A**). (**C**) Analysis of 2-APB-mediated outward currents from (**A**) at t = 178 s. (**D**) same experimental setting as in (**A**) but with 50 μM IP_3_ and 0 nM Ca^2+^ in the patch pipette (1-1-1-1, *n* = 9; 3-1-1-1, *n* = 13; 3-1-1-3, *n* = 6; 3-1-3-1, *n* = 11; 3-1-3-3, *n* = 8; control, *n* = 11). (**E**) Corresponding IV relationships from cells in (**D**). (**F**) Analysis of 2-APB-mediated outward currents from (**D**) at t = 178 s. Statistical significance was determined by an unpaired t test with Welch’s correction and is indicated as ns *p* > 0.05, * *p* < 0.05, ** *p* < 0.005, **** *p* < 0.0001.

We next transfected HEK 293 WT cells with 1-1-1-1, 3-1-1-1, 3-1-3-1, 3-1-1-3, 3-1-3-3 concatented channels or control and applied 50 µM 2-APB when intracellular Ca^2+^ was clamped to 200 nM (Figure 4A–C, IVs are shown in Figure 4B) or stores were depleted with IP_3_ and Ca^2+^ was clamped to near zero with 20 mM BAPTA (Figure 4D–F, IVs are shown in Figure 4E).

Similar to the experiments with HEK293 STIM1 cells (Figure 3C–E) the data in Figure 4 demonstrate that two or more Orai3 subunits in the concatenated channel are sufficient to mediate a 2-APB-induced outward current, when endogenous STIM1 is not recruited to the plasma membrane (Figure 4A–C) or may assemble with the channel (Figure 4D–F).

### 2.3. Comparison to Prostate Cancer Cells

In many cell types, e.g., T cells, B cells and mast cells, I_CRAC_ is first amplified and then completely blocked when 50 µM 2-APB is applied [41,42,43].

We have previously shown that in the prostate cancer cell line LNCaP a low number of Orai3 subunits within store-operated CRAC channels alters the 2-APB response compared to Orai1-mediated currents when 50 µM was applied [18].

**Figure 5:**
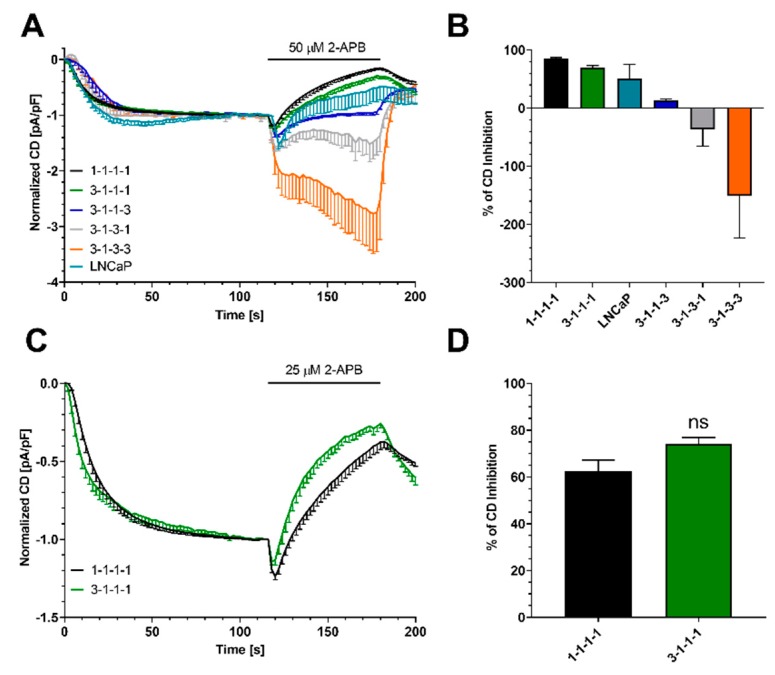
Normalized current density responses under store-depleting conditions in HEK cells stably overexpressing STIM1 with overexpression of concatenated tetrameric Orai1/Orai3 channels, and LNCaP prostate cancer cells. (**A**) CD development when 50 µM 2-APB was applied as indicated. Traces were normalized to the CD at t = 116 s after subtraction of leak currents (1-1-1-1, *n* = 4; 3-1-1-1, *n* = 5; 3-1-1-3, *n* = 5; 3-1-3-1, *n* = 6; 3-1-3-3, *n* = 5, LNCaP *n* = 14). (**B**) Percentage of 2-APB-mediated CD inhibition at t = 178 s extracted from (**A**). (**C**) CD development in concatenated constructs 1-1-1-1 and 3-1-1-1 (*n* = 7 and *n* = 9) when 25 µM 2-APB was applied as indicated. Traces were normalized to the CD at t = 116 s after leak subtraction. (**D**) Percentage of 2-APB-mediated CD inhibition at t = 178 s extracted from (**C**). Statistical significance in (**D**) was determined by an unpaired t test with Welch’s correction and is indicated as ns *p* > 0.05.

To compare data from LNCaP prostate cancer cells with concatenated channels, we normalized data from Figure 2 and data from LNCaP when 50 µM 2-APB were applied. Data were normalized to CD at 116 s when I_CRAC_ had fully developed (Figure 5A). A bar diagram of the 2-APB-induced block is shown in Figure 5B. We here omit all outward currents as outward current development was not observed in endogenous cells.

None of the concatenated channels displayed the exact pharmacological profile of LNCaP cells. However, I_CRAC_ from cells expressing concatenated channels exhibit a similar pharmacological profile as the prostate cancer cells. It is possible that the concatenation of subunits changes the complex pharmacological profile. Moreover, endogenous CRAC channels might exist as a mixed population of channels composed of varying numbers of Orai1/Orai3 subunits that cannot be simulated with one distinct channel composition.

Concatenated channels 1-1-1-1 and 3-1-1-1 gave similar results upon application of 50 µM 2-APB. Strikingly, one Orai3 subunit in 3-1-1-1 seemed to reduce the 2-APB-induced block compared to 1-1-1-1. We further evaluated this question by applying 25 µM 2-APB in a second experiment (Figure 5C,D). Under these conditions, we still detected the amplification of I_CRAC_ followed by a block. Adding one Orai3 subunit to the channel does not alter 2-APB pharmacological profile. Please note that under these conditions the 2-APB-induced block ([2-APB] = 30 µM) is absent in LNCaP cells [18].

Taken together, none of the concatenated channels displayed the exact pharmacological profile of LNCaP cells; however, here, we speculate that endogenous store-operated Orai1/Orai3 channels are formed with one or more Orai3 subunit(s).

## 3. Discussion

The versatile role of SOCE channels in physiology/pathophysiology make them an attractive therapeutic target. Endogenous heteromeric store-operated Orai/Orai3 channels exhibit a complex but promising pharmacology [16,18,25,27,28,40,59], however, their investigation is challenging. In this work we used concatenated channels with defined Orai1 and Orai3 subunit stoichiometry and arrangement to systematically investigate their complex pharmacology using a widely used tool compound (2-APB).

Upon activation by STIM1, all channels exhibited the typical inwardly rectified I_CRAC_. When store depletion was prevented or only endogenous STIM1 was present at the channel, no I_CRAC_ was observed. In line with previous studies [15,61], the expression of one or more Orai3 subunit(s) within these channels result in a significant reduction of I_CRAC_ that was also observed in endogenous systems [15,16,17,18,23,24]. In concatenated channels, expression of two or more Orai3 subunits resulted in 2-APB-induced outward currents in STIM1-dependent and store-independent 2-APB-activated channels. The amount of this outward current gradually increased with the number of Orai3 subunits (3-1-3-1 < 3-1-1-3 < 3-1-3-3). From experiments with channel constructs containing two Orai3 subunits, we can conclude that the pore size depends on the position of Orai1 and Orai3 subunits within the multimeric channel arrangement. To our knowledge, such outward currents were never observed in endogenous cells. Strikingly, presence of STIM1 at the channels increased 2-APB-induced outward component by 2-fold. This has been observed earlier [50] and may indicate that STIM1 is present at the 2-APB-activated channel and adds to ion channel conductivity. In contrast, it has been published that STIM1 dissociates from the channel before 2-APB-induced conductivity is observed [57]. One explanation might be that STIM1 activation before 2-APB application results in 2-APB-activated channels that differ from 2-APB-activated channels that originate from a closed (STIM1 free) state. In addition to STIM1/Orai1 and STIM1/Orai3, STIM2/Orai1 and STIM2/Orai3 can form store-operated channels. Without store depletion, STIM2/Orai1 overexpressing HEK293 cells give a transient current response upon application of 50 µM 2-APB that is absent in STIM1/Orai1-expressing cells [62]. Future studies may compare the 2-APB response from STIM2 and Orai1, Orai3, or concatenated channel overexpression systems to functionally analyze the pharmacology of STIM2-expressing endogenous cells.

The response of prostate cancer cells LNCaP to the application of 50 µM 2-APB could not be exactly simulated with any concatenated channel. Concatenated channels may differ in their response to 2-APB as subunits are linked by an additional intramolecular connection. In addition, endogenous channels may form mixed heteromeric populations in contrast to concatenated channels that represent one defined subunit composition. Furthermore, we cannot exclude that an additional 2-APB-sensitive component adds to heteromeric SOCE channels altering the Orai1/Orai3-mediated response such as TRPC [63,64].

The ROS-dependent block is abolished when one Orai3 subunit is present within the channel [15]. In contrast, no difference in current is observed between 1-1-1-1 and 3-1-1-1 in response to 2-APB. In conclusion, endogenous heteromeric Orai1/Orai3 channels with only one Orai3 subunit may be undistinguishable from homomeric Orai1 channels in their 2-APB profile. Development of tool compounds based on the known 2-APB or other scaffolds that distinguish homomeric Orai1 channels and heteromeric channels with one Orai3 subunit may help to identify heteromeric Orai channels in various endogenous cell types in the future. Blocking or activating distinct heteromeric store-operated channels may be the key for specific modulations of SOCE in cancer and immune therapy.

## 4. Materials and Methods

### 4.1. Cell Culture and Transfection

All cells were cultivated at 37 °C and 5% CO_2_. MEM medium (Gibco, Thermo Fisher Scientific, Waltham, MA, USA) supplemented with 10% FCS (Gibco) was used for HEK 293 WT cells. HEK 293 cells stably expressing STIM1 were grown in MEM medium supplemented with 10% FCS and 500 µg/mL G418 (Gibco). All cells were transfected with 1 µg DNA per 10^6^ cells in a Nucleofector 4D device with Nucleofector Kit SF (Lonza, Basel, Switzerland), according to the manufacturer’s guidelines. DNA constructs 1-1-1-1, 3-1-1-1, 3-1-3-1 and 3-1-3-3 were generated previously [33,65]. All work was performed under the ethics approval of the Bundesamt für Umwelt, Switzerland to the Institute of Biochemistry and Molecular Medicine (A141370).

### 4.2. Electrophysiology

All patch-clamp experiments were performed in a whole-cell setting at 20-24 °C. Currents were acquired and filtered at 2.9 kHz with a HEKA EPC-10 amplifier (HEKA Elektronik, Lambrecht (Pfalz), Germany) and recorded with the HEKA Patchmaster (v2×53) software. Every 2 s, 50 ms voltage ramps spanning −150 mV to 150 mV from a holding potential of 0 mV were applied. All currents were corrected for a liquid junction potential of 10 mV. Capacitive currents were corrected before every voltage ramp delivery. For analysis, currents at −130 mV and 130 mV were extracted, normalized to cell capacitance, and plotted versus time. The error bars represent the standard error of the mean (SEM) if not indicated otherwise. Any additional normalizations, if used, are mentioned in the figure captions. Data was analyzed with Igor Pro 6.37 (Wavemetrics, Lake Oswego, OR, USA). Bath solution contained: 120 mM NaCl, 20 mM CaCl_2_, 10 mM TEA-Cl, 10 mM HEPES, 2 mM MgCl_2_. Store-depleting pipette solution contained: 120 mM Cs-glutamate, 20 mM BAPTA (10 mM for LNCaP), 10 mM HEPES, 3 mM MgCl_2_, 50 µM IP_3._ In pipette solution that prevented store depletion, IP_3_ was omitted and calcium was clamped to 200 nM with BAPTA. Calcium concentration was calculated according to WEBMAXC STANDARD [66]. Osmolarity was adjusted with glucose to 310 mOsm. Under these conditions, pipettes had resistances of 2–3 MΩ.

### 4.3. Statistical Analysis

Statistical tests were performed in GraphPad Prism 8 (GraphPad Software, San Diego, CA, USA) as detailed in the figure captions.

## 5. Conclusions

Targeting endogenous heteromeric ion channels in therapeutic interventions is generally very challenging. Often their pharmacological behavior varies with subunit composition and arrangement. Here, we used concatenated SOCE channels in order to rationalize the action of 2-APB on different Orai1/Orai3 channel architectures. Our findings show that as Orai3 content is increased, in particular, 2-APB pharmacology digresses significantly from mostly Orai1 containing channels. Ultimately, our long-term goal is to generate SOCE channel subtype-dependent pharmacology profiles that can be exploited therapeutically in diseased native cells.

## Abbreviations

IP_3_	inositol 1,4,5-trisphosphate
SOCE	store-operated Ca^2+^ entry
I_CRAC_	Ca^2+^ release activated Ca^2+^ current
Ca^2+^	calcium ions

## References

[1] Demuro A., Penna A., Safrina O., Yeromin A.V., Amcheslavsky A., Cahalan M.D., Parker I. (2011). Subunit stoichiometry of human Orai1 and Orai3 channels in closed and open states. Proc. Natl. Acad. Sci. USA.

[2] Ji W., Xu P., Li Z., Lu J., Liu L., Zhan Y., Chen Y., Hille B., Xu T., Chen L. (2008). Functional stoichiometry of the unitary calcium-release-activated calcium channel. Proc. Natl. Acad. Sci. USA.

[3] Penna A., Demuro A., Yeromin A.V., Zhang S.L., Safrina O., Parker I., Cahalan M.D. (2008). The CRAC channel consists of a tetramer formed by Stim-induced dimerization of Orai dimers. Nature.

[4] Park C.Y., Hoover P.J., Mullins F.M., Bachhawat P., Covington E.D., Raunser S., Walz T., Garcia K.C., Dolmetsch R.E., Lewis R.S. (2009). STIM1 clusters and activates CRAC channels via direct binding of a cytosolic domain to Orai1. Cell.

[5] Hou X., Pedi L., Diver M.M., Long S.B. (2012). Crystal structure of the calcium release-activated calcium channel orai. Science.

[6] Schmidt B., Alansary D., Bogeski I., Niemeyer B.A., Rieger H. (2019). Reaction-diffusion model for STIM-ORAI interaction: The role of ROS and mutations. J. Theor. Biol..

[7] Yen M., Lokteva L.A., Lewis R.S. (2016). Functional analysis of orai1 concatemers supports a hexameric stoichiometry for the CRAC channel. Biophys. J..

[8] Alansary D., Peckys D.B., Niemeyer B.A., de Jonge N. (2020). Detecting single ORAI1 proteins within the plasma membrane reveals higher-order channel complexes. J. Cell Sci..

[9] Hoover P.J., Lewis R.S. (2011). Stoichiometric requirements for trapping and gating of Ca2+ release-activated Ca2+ (CRAC) channels by stromal interaction molecule 1 (STIM1). Proc. Natl. Acad. Sci. USA.

[10] Kilch T., Alansary D., Peglow M., Dörr K., Rychkov G., Rieger H., Peinelt C., Niemeyer B.A. (2013). Mutations of the Ca2+-sensing stromal interaction molecule STIM1 Regulate Ca2+ Influx by altered oligomerization of STIM1 and by destabilization of the Ca2+ channel Orai1. J. Biol. Chem..

[11] Li Z., Liu L., Deng Y., Ji W., Du W., Xu P., Chen L., Xu T. (2011). Graded activation of CRAC channel by binding of different numbers of STIM1 to Orai1 subunits. Cell Res..

[12] Yen M., Lewis R.S. (2019). Numbers count: How STIM and Orai stoichiometry affect store-operated calcium entry. Cell Calcium.

[13] Gwack Y., Srikanth S., Feske S., Cruz-Guilloty F., Oh-hora M., Neems D.S., Hogan P.G., Rao A. (2007). Biochemical and functional characterization of Orai proteins. J. Biol. Chem..

[14] Lis A., Peinelt C., Beck A., Parvez S., Monteilh-Zoller M., Fleig A., Penner R. (2007). CRACM1, CRACM2, and CRACM3 are Store-Operated Ca2+ channels with distinct functional properties. Curr. Biol..

[15] Alansary D., Bogeski I., Niemeyer B.A. (2015). Facilitation of Orai3 targeting and store-operated function by Orai1. Biochim. Biophys. Acta.

[16] Bogeski I., Kummerow C., Al-Ansary D., Schwarz E.C., Koehler R., Kozai D., Takahashi N., Peinelt C., Griesemer D., Bozem M. (2010). Differential redox regulation of ORAI ion channels: A mechanism to tune cellular calcium signaling. Sci. Signal..

[17] Eckstein M., Vaeth M., Aulestia F.J., Costiniti V., Kassam S.N., Bromage T.G., Pedersen P., Issekutz T., Idaghdour Y., Moursi A.M. (2019). Differential regulation of Ca2+ influx by ORAI channels mediates enamel mineralization. Sci. Signal..

[18] Holzmann C., Kilch T., Kappel S., Armbrüster A., Jung V., Stöckle M., Bogeski I., Schwarz E.C., Peinelt C. (2013). ICRAC controls the rapid androgen response in human primary prostate epithelial cells and is altered in prostate cancer. Oncotarget.

[19] Holzmann C., Kilch T., Kappel S., Dörr K., Jung V., Stöckle M., Bogeski I., Peinelt C. (2015). Differential redox regulation of Ca^2+^ signaling and viability in normal and malignant prostate cells. Biophys. J..

[20] Kwon J., An H., Sa M., Won J., Shin J.I., Lee C.J. (2017). Orai1 and Orai3 in combination with Stim1 mediate the majority of store-operated calcium entry in astrocytes. Exp. Neurobiol..

[21] Vaeth M., Yang J., Yamashita M., Zee I., Eckstein M., Knosp C., Kaufmann U., Karoly Jani P., Lacruz R.S., Flockerzi V. (2017). ORAI2 modulates store-operated calcium entry and T cell-mediated immunity. Nat. Commun..

[22] Angenendt A., Steiner R., Knörck A., Schwär G., Konrad M., Krause E., Lis A. (2020). Orai, STIM, and PMCA contribute to reduced calcium signal generation in CD8^+^ T cells of elderly mice. Aging (Albany N. Y.).

[23] Kilch T., Kappel S., Peinelt C. (2016). Regulation of Ca(2+) signaling in prostate cancer cells. Channels (Austin).

[24] Saul S., Gibhardt C.S., Schmidt B., Lis A., Pasieka B., Conrad D., Jung P., Gaupp R., Wonnenberg B., Diler E. (2016). A calcium-redox feedback loop controls human monocyte immune responses: The role of ORAI Ca2+ channels. Sci. Signal..

[25] Ay A.-S., Benzerdjeb N., Benzerdjerb N., Sevestre H., Ahidouch A., Ouadid-Ahidouch H. (2013). Orai3 constitutes a native store-operated calcium entry that regulates non small cell lung adenocarcinoma cell proliferation. PLoS ONE.

[26] Faouzi M., Hague F., Potier M., Ahidouch A., Sevestre H., Ouadid-Ahidouch H. (2011). Down-regulation of Orai3 arrests cell-cycle progression and induces apoptosis in breast cancer cells but not in normal breast epithelial cells. J. Cell. Physiol..

[27] Motiani R.K., Abdullaev I.F., Trebak M. (2010). A novel native store-operated calcium channel encoded by Orai3: Selective requirement of Orai3 versus Orai1 in estrogen receptor-positive versus estrogen receptor-negative breast cancer cells. J. Biol. Chem..

[28] Wei D., Mei Y., Xia J., Hu H. (2017). Orai1 and Orai3 mediate Store-Operated calcium entry contributing to neuronal excitability in dorsal root ganglion neurons. Front. Cell. Neurosci..

[29] Dubois C., Vanden Abeele F., Lehen’kyi V., Gkika D., Guarmit B., Lepage G., Slomianny C., Borowiec A.S., Bidaux G., Benahmed M. (2014). Remodeling of channel-forming ORAI proteins determines an oncogenic switch in prostate cancer. Cancer Cell.

[30] González-Cobos J.C., Zhang X., Zhang W., Ruhle B., Motiani R.K., Schindl R., Muik M., Spinelli A.M., Bisaillon J.M., Shinde A.V. (2013). Store-Independent Orai1/3 channels activated by intracrine leukotrieneC_4_. Circ. Res..

[31] Zhang X., Zhang W., González-Cobos J.C., Jardin I., Romanin C., Matrougui K., Trebak M. (2014). Complex role of STIM1 in the activation of store-independent Orai1/3 channels. J. Gen. Physiol..

[32] Thompson J.L., Mignen O., Shuttleworth T.J. (2013). The ARC Channel—An Endogenous Store-Independent Orai Channel. Current Topics in Membranes.

[33] Mignen O., Thompson J.L., Shuttleworth T.J. (2009). The molecular architecture of the arachidonate-regulated Ca ^2+^ -selective ARC channel is a pentameric assembly of Orai1 and Orai3 subunits. J. Physiol..

[34] Leon-Aparicio D., Pacheco J., Chavez-Reyes J., Galindo J.M., Valdes J., Vaca L., Guerrero-Hernandez A. (2017). Orai3 channel is the 2-APB-induced endoplasmic reticulum calcium leak. Cell Calcium.

[35] Benzerdjeb N., Sevestre H., Ahidouch A., Ouadid-Ahidouch H. (2016). Orai3 is a predictive marker of metastasis and survival in resectable lung adenocarcinoma. Oncotarget.

[36] Denda S., Takei K., Kumamoto J., Goto M., Denda M. (2017). Expression level of Orai3 correlates with aging-related changes in mechanical stimulation-induced calcium signalling in keratinocytes. Exp. Dermatol..

[37] Faouzi M., Kischel P., Hague F., Ahidouch A., Benzerdjeb N., Sevestre H., Penner R., Ouadid-Ahidouch H. (2013). ORAI3 silencing alters cell proliferation and cell cycle progression via c-myc pathway in breast cancer cells. Biochim. Biophys. Acta Mol. Cell Res..

[38] Hasna J., Hague F., Rodat-Despoix L., Geerts D., Leroy C., Tulasne D., Ouadid-Ahidouch H., Kischel P. (2018). Orai3 calcium channel and resistance to chemotherapy in breast cancer cells: The p53 connection. Cell Death Differ..

[39] Kappel S., Marques I.J., Zoni E., Stokłosa P., Peinelt C., Mercader N., Kruithof-de Julio M., Borgström A. (2017). Store-Operated Ca2+ entry as a prostate cancer biomarker—A riddle with perspectives. Curr. Mol. Biol. Rep..

[40] Motiani R.K., Zhang X., Harmon K.E., Keller R.S., Matrougui K., Bennett J.A., Trebak M. (2013). Orai3 is an estrogen receptor α-regulated Ca ^2+^ channel that promotes tumorigenesis. FASEB J..

[41] Kukkonen J.P., Lund P.-E., Åkerman K.E.O. (2001). 2-aminoethoxydiphenyl borate reveals heterogeneity in receptor-activated Ca2+discharge and store-operated Ca2+influx. Cell Calcium.

[42] Prakriya M., Lewis R.S. (2001). Potentiation and inhibition of Ca ^2+^ release-activated Ca ^2+^ channels by 2-aminoethyldiphenyl borate (2-APB) occurs independently of IP _3_ receptors. J. Physiol..

[43] Ma H.-T., Venkatachalam K., Parys J.B., Gill D.L. (2002). Modification of store-operated channel coupling and inositol trisphosphate receptor function by 2-aminoethoxydiphenyl borate in DT40 lymphocytes. J. Biol. Chem..

[44] Peinelt C., Vig M., Koomoa D.L., Beck A., Nadler M.J.S., Koblan-Huberson M., Lis A., Fleig A., Penner R., Kinet J.-P. (2006). Amplification of CRAC current by STIM1 and CRACM1 (Orai1). Nat. Cell Biol..

[45] Scrimgeour N., Litjens T., Ma L., Barritt G.J., Rychkov G.Y. (2009). Properties of Orai1 mediated store-operated current depend on the expression levels of STIM1 and Orai1 proteins. J. Physiol..

[46] Hendron E., Wang X., Zhou Y., Cai X., Goto J., Mikoshiba K., Baba Y., Kurosaki T., Wang Y., Gill D.L. (2014). Potent functional uncoupling between STIM1 and Orai1 by dimeric 2-aminodiphenyl borinate analogs. Cell Calcium.

[47] Xu X., Ali S., Li Y., Yu H., Zhang M., Lu J., Xu T. (2016). 2-Aminoethoxydiphenyl borate potentiates CRAC current by directly dilating the pore of open orai1. Sci. Rep..

[48] Wei M., Zhou Y., Sun A., Ma G., He L., Zhou L., Zhang S., Liu J., Zhang S.L., Gill D.L. (2016). Molecular mechanisms underlying inhibition of STIM1-Orai1-mediated Ca2+ entry induced by 2-aminoethoxydiphenyl borate. Pflug. Arch..

[49] De Haven W.I., Smyth J.T., Boyles R.R., Bird G.S., Putney J.W. (2008). Complex actions of 2-aminoethyldiphenyl borate on store-operated calcium entry. J. Biol. Chem..

[50] Peinelt C., Lis A., Beck A., Fleig A., Penner R. (2008). 2-Aminoethoxydiphenyl borate directly facilitates and indirectly inhibits STIM1-dependent gating of CRAC channels. J. Physiol..

[51] Zhang S.L., Kozak J.A., Jiang W., Yeromin A.V., Chen J., Yu Y., Penna A., Shen W., Chi V., Cahalan M.D. (2008). Store-dependent and -independent modes regulating Ca ^2+^ Release-activated Ca ^2+^ channel activity of human Orai1 and Orai3. J. Biol. Chem..

[52] Schindl R., Bergsmann J., Frischauf I., Derler I., Fahrner M., Muik M., Fritsch R., Groschner K., Romanin C. (2008). 2-aminoethoxydiphenyl borate alters selectivity of Orai3 channels by increasing their pore size. J. Biol. Chem..

[53] Amcheslavsky A., Safrina O., Cahalan M.D. (2013). Orai3 TM3 point mutation G158C alters kinetics of 2-APB-induced gating by disulfide bridge formation with TM2 C101. J. Gen. Physiol..

[54] Amcheslavsky A., Safrina O., Cahalan M.D. (2014). State-dependent block of Orai3 TM1 and TM3 cysteine mutants: Insights into 2-APB activation. J. Gen. Physiol..

[55] Bergsmann J., Derler I., Muik M., Frischauf I., Fahrner M., Pollheimer P., Schwarzinger C., Gruber H.J., Groschner K., Romanin C. (2011). Molecular determinants within N terminus of Orai3 protein that control channel activation and gating. J. Biol. Chem..

[56] Kar P., Samanta K., Kramer H., Morris O., Bakowski D., Parekh A.B. (2014). Dynamic assembly of a membrane signaling complex enables selective activation of NFAT by Orai1. Curr. Biol..

[57] Yamashita M., Somasundaram A., Prakriya M. (2011). Competitive modulation of Ca2+ release-activated Ca2+ channel gating by STIM1 and 2-aminoethyldiphenyl borate. J. Biol. Chem..

[58] Schindl R., Frischauf I., Bergsmann J., Muik M., Derler I., Lackner B., Groschner K., Romanin C. (2009). Plasticity in Ca2+ selectivity of Orai1/Orai3 heteromeric channel. Proc. Natl. Acad. Sci. USA.

[59] Bhattacharya A., Kumar J., Hermanson K., Sun Y., Qureshi H., Perley D., Scheidegger A., Singh B.B., Dhasarathy A., Bhattacharya A. (2018). The calcium channel proteins ORAI3 and STIM1 mediate TGF-β induced Snai1 expression. Oncotarget.

[60] Bogeski I., Al-Ansary D., Qu B., Niemeyer B.A., Hoth M., Peinelt C. (2010). Pharmacology of ORAI channels as a tool to understand their physiological functions. Expert Rev. Clin. Pharmacol..

[61] Mignen O., Thompson J.L., Shuttleworth T.J. (2008). Both Orai1 and Orai3 are essential components of the arachidonate-regulated Ca ^2+^ -selective (ARC) channels. J. Physiol..

[62] Parvez S., Beck A., Peinelt C., Soboloff J., Lis A., Monteilh-Zoller M., Gill D.L., Fleig A., Penner R. (2008). STIM2 protein mediates distinct store-dependent and store-independent modes of CRAC channel activation. FASEB J..

[63] Hong J.H., Li Q., Kim M.S., Shin D.M., Feske S., Birnbaumer L., Cheng K.T., Ambudkar I.S., Muallem S. (2011). Polarized but Differential Localization and Recruitment of STIM1, Orai1 and TRPC Channels in Secretory Cells. Traffic.

[64] Cheng K.T., Ong H.L., Liu X., Ambudkar I.S. (2013). Contribution and regulation of TRPC channels in store-operated Ca2+ Entry. Current Topics in Membranes.

[65] Mignen O., Thompson J.L., Shuttleworth T.J. (2008). Orai1 subunit stoichiometry of the mammalian CRAC channel pore. J. Physiol..

[66] WEBMAXC STANDARD. https://somapp.ucdmc.ucdavis.edu/pharmacology/bers/maxchelator/webmaxc/webmaxcS.htm.

